# Dirac fermions duality in graphene: Ripples and fractional dimensions as function of temperature

**DOI:** 10.1038/s41598-018-31944-y

**Published:** 2018-11-02

**Authors:** J. C. Flores, L. Palma-Chilla

**Affiliations:** 1 Instituto de Alta investigación IAI, Universidad ded Tarapacá, Casilla 7-D, Arica, Chile; 2 Departamento de Física, Universidad de La Serena, Av-Juan Cisternas, 1200 La Serena, Chile

## Abstract

Graphene consists of coupled direct/dual fermionic sub-systems and, consequently, the thermal properties of both are intrinsically correlated. The dual is characterized by negative temperatures, and its free energy keeps opposite sign concerning the direct. The growth of ripples in graphene becomes related to temperature rises with fractional spatial dimension ~2.19 at 300 °K. An analytical, and suitable, expression for ripples dimension as a function of temperature is presented. Further, internal energy, entropy, specific heat and free energy are evaluated as a function of temperature and dimension for both sub-systems. Free energy supports a simple, functional expression inversely proportional to ripples dimension.

## Introduction

For a normal direct system with non decreasing frequency $\omega (\overrightarrow{k})$ its dual of frequency *ω*′ (behaving usually as a metamaterial) is characterized through a functional relationship^1–5^. Particularly, we consider the relationship:

1 $$\omega ^{\prime} (\overrightarrow{k})+\omega (\overrightarrow{k})=2{\omega }_{o}$$

*ω*_*o*_ being an intrinsic parameter. Equation (1) can be found in a large variety of systems like electric circuits, electron/hole, and others. Indeed, massless fermions in graphene, which have two bands in the range of discussion, justly satisfied Eq. (1) with *ω*_*o*_ = 0, assumed in this article. Additionally, the dual system properties will always be indexed with a prime (like *ω*′).

If we designate *U* as the internal thermodynamic energy per particle, from Eq. (1), entropy *S*′ of the dual system becomes related to entropy *S* of the direct by

2 $$S^{\prime} (U)=S(\,-\,U).$$

This way, and formally, all thermodynamic properties of the dual system are obtained from the direct system via Eq. (2). Particularly, temperature^6–8^ *T* = ∂*U*/∂*S* turns negative for the dual.

At first order in the wave vector expansion, for a graphene sheet or carbon monolayer, the “massless” electrons spectrum is given by^9–12^

3 $$\hslash \omega =\hslash {v}_{F}|\overrightarrow{k}|$$

*v*_*F*_ ~ 10^6^ (m/s) being the Fermi velocity and $\hslash$ the Planck constant. Formally, Eq. (3) defines a hypersphere in the wavevector space. This becomes useful for calculating the number of states at fixed energy. Additionally, for rippleless graphene the spatial dimension is exactly *D* = 2.

On the other hand, relationships (1) and (3) define the dual system which consequently has a dispersion relationship

4 $$\hslash \omega ^{\prime} =-\,\hslash {v}_{F}|\overrightarrow{k}|$$

actually corresponding to the graphene lower band.

In this paper, non-integer spatial dimension is mainly considered since a graphene sheet with aleatory broken bonds, ribbons, ripples, or others, can be modeled by a fractional dimension *D* ~ 2. We shall consider thermodynamic aspects and, particularly, the fractional dimension due to ripples related to finite temperature^11,13^.

Section II establishes the entropy function for the massless electrons in the upper band. In section III, employing the direct and dual entropy relationship Eq. (2), all thermodynamic quantities for the corresponding Dirac fermions are calculated. They are expressed explicitly as a function of dimension and temperature. Main results appear in section IV where we evaluate an analytical expression for the spatial ripples dimension of graphene as a function of temperature. The last section offers the conclusions.

## Direct System: Electrons in Graphene

As long as Fermi energy is zero for fermions in graphene^14^, from the hypersphere defined by Eq. (3), at low temperature the internal energy for electrons can be expressed as

5 $$\frac{U}{{U}_{o}}=\sigma {(\frac{kT}{{U}_{o}})}^{D+1}$$

where *U*_*o*_ = ℏ*v*_*F*_/*a* ~ 7.4296 × 10^−19^ (J), *k* is the Boltzmann constant and the length *a* ~ 1.42 (A) corresponds to the approximate distance between carbon atoms in the hexagonal cell^14^. *σ* is a function of spatial dimension *D* with spin-degeneration two and given by

6 $$\sigma (D)=2\times \frac{D{\pi }^{D/2}}{{\rm{\Gamma }}(D/2+1)}(\frac{1}{{2}^{D}})\,{\int }_{0}^{\infty }\,\frac{{x}^{D}}{{e}^{x}+1}dx$$

**Figure 1:**
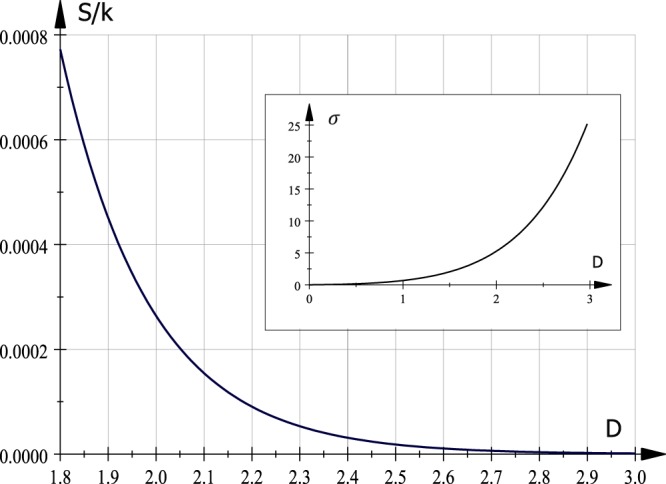
Entropy *S*/*k*, per particle, for direct and dual systems in graphene as function of dimension *D* at *T* = ±300 °K. Inset, the parameter *σ* as function of spatial dimension *D*. It allows to evaluate thermodynamic properties around *D* = 2 where *σ* = 5.659.

Figure 1 exhibits a numerical calculation of *σ* as function of *D*. At dimension *D* = 2 its value corresponds to *σ* = 5.659. This function will be used to evaluate thermodynamic properties around *D* ~ 2.

The entropy as a function of the internal energy can be obtained as follows. From Eq. (5) and the usual definition for temperature^6–8^ (∂*S*/∂*U* = 1/*T*) the differential equation

7 $$\frac{dS}{dU}=\frac{k}{{U}_{o}}{(\frac{U}{\sigma {U}_{o}})}^{\frac{1}{D+1}}$$

is attained. We put total derivative since the volume is assumed constant. The solution of this differential equation, with the appropriate initial condition *S* = 0 when *U* = 0 allows to determine the entropy *per* particle for the direct system as

8 $$S(U)=k\frac{D+1}{D}{\sigma }^{\frac{1}{D+1}}{(\frac{aU}{\hslash {v}_{F}})}^{\frac{D}{D+1}};\,U\ge 0$$

where, as mentioned, *U*_*o*_ = ℏ*v*_*F*_/*a* for graphene. In Eq. (8) we use *a* instead of total size to avoid Gibbs paradox.

## Thermodynamic Relationships for Direct/Dual Dirac Fermions and Dimension Dependences

From Eq. (2) and the entropy Eq. (8) the main properties can be calculated for both interrelated systems as a function of dimension and temperature. They appear in Table 1, showing internal energy *U*, entropy *S*, specific heat *C* and free energy *F*.

**Table 1:** Thermal properties of direct (electrons) and dual (holes) massless fermions in graphene at arbitrary dimension and temperature. Internal energy, entropy, specific heat and free energy, are showed.

	Direct(*T* ≥ 0)	Dual(*T* ≤ 0)
*U*/*σ*	$\frac{\hslash {v}_{F}}{a}{(\frac{kTa}{\hslash {v}_{F}})}^{D+1}$	$-\,\frac{\hslash {v}_{F}}{a}{(-\frac{kTa}{\hslash {v}_{F}})}^{D+1}$
*S*/*kσ*	$\frac{D+1}{D}{(\frac{kTa}{\hslash {v}_{F}})}^{D}$	$\frac{D+1}{D}{(-\frac{kTa}{\hslash {v}_{F}})}^{D}$
*C*/*kσ*	$(D+\mathrm{1)}{(\frac{akT}{\hslash {v}_{F}})}^{D}$	$(D+\mathrm{1)}{(-\frac{akT}{\hslash {v}_{F}})}^{D}$
*F*/*σ*	$-\,\frac{1}{D}\frac{\hslash {v}_{F}}{a}{(\frac{kTa}{\hslash {v}_{F}})}^{D+1}$	$\frac{1}{D}\frac{\hslash {v}_{F}}{a}{(-\frac{kTa}{\hslash {v}_{F}})}^{D+1}$

Note that temperature turns negative in the dual system. Moreover, free energy *F* = *U* − *TS* can be re-written in both cases as a function of internal energy and dimension simply as

9 $$F=-\,\frac{U}{D}.$$

Both direct/dual free energy *F* have opposite sign because of *U*. Likewise, the free-energy-cost parameter ∂*F*/∂*D* = *U*/*D*^2^ (*U* fixed).

The graphic for the graphene entropy as a function of the dimension appears in Fig. 1 at fixed temperature (*T* = ±300 °K.). It is a decreasing function since dimensionless temperature *kTa*/ℏ*v*_*F*_ ~ 5.5749 × 10^−3^ (powered at *D*, Table 1) is a small number.

## Spatial Dimension as Function of Temperature: Ripples in Graphene

Graphene seems to be a real two-dimensional system^11,12,15^. Yet, this spatial dimension varies slightly depending for instance on imperfections like bad bonds, rugosity or others^11,16,17^. In fact, distortions affect transport properties like electronic mobility^17–20^. Specifically, transport becomes affected by rising temperature^21,22^. Additionally, decoherence effects, due to temperature, also affect conduction.

For example, Giordanelli *et al*.^13^ consider ripples for graphene sheets and find a fractional dimension at the order of 1.16 for islands in an iso-height plane at the percolation threshold. Since it corresponds to the intersection of a plane with the rippled graphene, fractional dimension *D* is at that point given by equation: 2 + *D* = 3 + 1.16. Namely, *D* ~ 2.16.

Ripples are associated with soft structures particularly with instabilities at dimension two^23^ where they range between 2 ≤ *D* ≤ 3. These structure distortions can be related to thermal processes^23–26^ among other causes^11^. The microscopic relationships (3–4) define two hyperspheres in the phase-space. Consequently, their volume ~ *U*^*D*^ and necessarily *T* ~ *U* in the microcanonical ensemble for direct and dual fermions. As long as the number of states becomes proportional to the volume^6–8^ and, consequently, to *T*^*D*^, the relationship between thermodynamic averaged-dimension 〈*D*〉 and temperature *T* becomes.

10 $$\langle D\rangle =\frac{{\int }_{2}^{3}\,dD[D][{(k|T|a/\hslash {v}_{F})}^{D}]}{{\int }_{2}^{3}\,dD[{(k|T|a/\hslash {v}_{F})}^{D}]}.$$

Its analytic solution is

11 $$\langle D\rangle =(\frac{3(k|T|a/\hslash {v}_{F})-2}{k|T|a/\hslash {v}_{F}-1})-\frac{1}{\mathrm{ln}(k|T|a/\hslash {v}_{F})}.$$

The formal limit 〈*D*〉 → 2 requires *T* → 0. Conversely, when *T* → ±∞ necessarily 〈*D*〉 → 3. In fact, these limits are governed by the slow logarithm function as 〈*D*〉 ~ 2 − 1/ln|*T*| and 〈*D*〉 ~ 3 − 1/ln|*T*| respectively.

**Figure 2:**
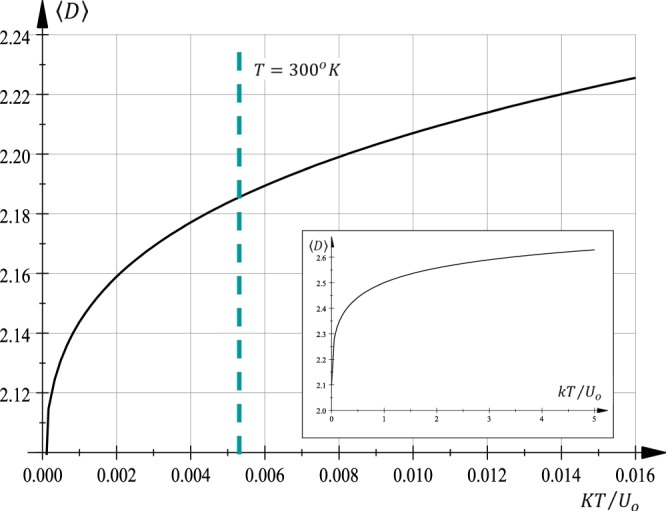
Graphene spatial averaged dimension 〈*D*〉 as function of dimensionless temperature *kT*/*U*_*o*_, where *U*_0_ = ℏ*v*_*F*_/*a*. For 300 °K the fractional dimension becomes ~2.1872 (vertical dashed line). Inset, a graph showing the tendencies for dimension as a function of temperature. In fact, 〈*D*〉 → 2 when *T* → 0 and 〈*D*〉 → 3 when *T* → ∞.

Figure 2 shows the average dimension 〈*D*〉 as function of the dimensionless temperature *kTa*/ℏ*v*_*F*_ for graphene around *T* = 300 (°*K*). In fact, the dimension of ripples distortion grows with temperature (Fig. 2 inset). Additionally, as long as broken bonds, ribbons, and ripples are manifestations of disorder (loss of translation invariance) they can be related, when exist, to general entropy concepts. Consequently, these inhomogeneous structures become correlated to a fractal dimension.

An explicit calculation at *T* = 300 (°*K*), gives the estimation for dimensionless temperature *kTa*/ℏ*v*_*F*_ ~ 0.0056 and, consequently, from^11^ the averaged dimension (Fig. 2):

12 $$\langle D\rangle \sim 2.1872\,({\rm{at}}\,T=300\,^\circ {\rm{K}})$$

firmly in line with Giordanelli *et al*.^13^ for graphene island intersected with iso-height plane (2.1872 − 1). The value of 300 °K corresponds to 28 °C (room temperature) and the above result Eq. (12) corresponds to an independent prediction. Prospectus to measure ripples for a broad range of temperatures were considered by Braghin and Hasselmann^27^.

Dimension fluctuations are evaluated directly. From Eq. (11) around *T* = 300 (°*K*) we have ${\rm{\Delta }}D\sim 6\times 0.0056\frac{{\rm{\Delta }}T}{T}$. Interestingly, for temperature ranges |Δ*T*| = 300 (°K) there are small variations Δ*D* ~ 0.0336 and not contradictory with Giordanelli *et al*.^13^.

Finally, note that Hurst’s exponent is related to the existence of different degrees of correlations in a given “roughness” structure. Indeed there is, with certain assumptions, a direct relation between this exponent and the fractal dimension^18,28,29^. From this point of view, as long as the fractional dimension can be calculated from thermodynamics, the Hurst’s exponent, eventually, can also be computed. On the other hand, quantities like curvature^13^, averaged height and disorder^30^, among others, also characterize the graphene structure and topology. Like as occur with Hurst’s exponent, these quantities are expected to be also correlated with temperature.

## Conclusions

Duality is quantitatively associated with graphene through the upper and lower bands (Eq. (1)). In this way, the internal energy, entropy, specific heat and free energy for direct/dual Dirac fermions were obtained as a function of the spatial dimension and temperature (Table 1). Remarkably, free energy supports a simple functional expression depending only on dimension and internal energy Eq. (9). Note that the dual and direct, related to upper and lower band, have different temperature sign. Then, they cannot be in equilibrium without external forces or constraints. They must be deeply related to collapse of pair electron-hole. As an interesting remark, both structures, dual and direct, admit *T* = 0 as commune point of equilibrium.

The dual system becomes characterized by negative temperatures and its free energy possesses opposite sign concerning the direct one. Moreover, at fixed temperature *T* = ±300 °K, the entropy decreases with dimensions augmentation since the effective temperature |*kTa*/ℏ*v*_*F*_| (powered at *D*) becomes a small number.

Regarding ripples, our analytical expression for dimension Eq. (10) as function of temperature allows estimating a non-integer dimension around 2.1872 and small fluctuations Δ*D* ~ 0.0336 at *T* = 300 °K. This fractional dimension augments as temperature rises.

Additionally, bilayered graphene model by putting *ω*_*o*_ ≠ 0 in Eq. (1) can be studied by using the direct-dual concepts^31^.
